# A Comparative View of the Principal Theories Which Have Prevailed in Chemistry Since the Beginning of the Present Century

**Published:** 1799-03

**Authors:** 

**Affiliations:** Sen. of Vienna


### *A comparative View of the principal Theories which have prevailed in Chemiſtry ſince the beginning of the preſent Century, By* Dr. Frank, *Sen. of Vienna.*

THERE is no branch of uſeful and intereſting knowledge, which as to its *practical tendency*, has been ſo long and unaccountably neglected by the bulk of mankind, and which at the ſame time has experienced ſo many whimſical revolutions in doctrine, and miſapplications in experiment, as that of chemiſtry.

Previous to the time of the learned Becher *, this rational and truly philoſophical

*John Joachim Becher, born in 1645, at Spires in Germany, was firſt Profeſſor of medicine, and then firſt phyſician to the Elector of Mentz, and afterwards to philoſophical ſcience was much upon a level with aſtrology; the chemiſts of thoſe ages were, in fact, little better than frantic alchemiſts, ſordid adventurers after what they termed the philoſopher's ſtone, or in other words, downright enthuſiaſts, who expected to prolong their ill-ſpent lives, and to accumulate immenſe wealth, by means of alchemical reſearches and productions.

Beccher ſurpaſſed both his cotemporaries and predeceſſors, in a very eminent degree: his obſervations were directed with great acuteneſs and accuracy to the nature and origin of the phenomena occurring in his chemical laboratory, and he was the firſt who, in this ſcience, attempted to combine theory with practice. It cannot however be aſſerted of him with truth, that he was not affected with the epidemic mania of the times; on the contrary, the chimerical and childiſh purſuit of converting baſe metals into gold, was his principal ſtudy, occupation, and probably the prediſpoſing inducement with him, to exert himſelf in his various phyſical reſearches, to penetrate more deeply into the nature of corporeal ſubſtances. Hence even the uſeful part of his doctrine is ſo interſperſed and obſcured with alchemical conjectures, that it becomes a matter of difficulty to ſeparate the droſs from the ore; ſo much do his works abound with theſe excreſcences of a fertile imagination!—At the time when this ſemi-philoſopher ſhone as a luminary in chemiſtry, there aroſe a man whoſe luminous, comprehenſive mind, contraſted with the puny ideas of contemporary writers, ſtill claims our reſpectful notice and admiration. It will be readily apprehended, that we allude here to the celebrated STAHL *; a man raiſed up to illuſtrate and eſtabliſh genuine chemiſtry, to the elector of Bavaria. He came to London, where he found an asylum from the malice of his invidious enemies, and died here in 1685. Besides various chemical and alchemical works, he published a book intitled, "*Character pro notitiâ linguarum universali:*" i. e. A universal language, by the medium of which all nations might understand each other. It is the fanciful idea of a man of genius; although it is not improbable that he was indebted for the first hint, on this curious subject, to the cotemporary work of Bishop WILKINS, published in 1668, folio: "*An Essay towards a real Character and a philosophical language.*" Beccher was the first who applied chemistry in all its branches to philosophy, and showed what use might be made of it, in explaining the structure, the combinations, and the mutual relations of bodies. He pretended to have invented a sort of perpetual motion, as he was a very able machinist. The world is indebted to him for several useful discoveries, and he also attempted to make some improvement in the art of printing.

* George Ernest Stahl, was born in Franconia, in 1660, and chosen Professor of medicine at Halle, when that celebrated university was founded in 1694. The excellency of his lectures, while he filled that chair; the importance of his various publications and his extensive practice, soon raised his reputation to a great height. He received an invitation to settle at Berlin, which he accepted, and was made counsellor of state and physician to the king. He died in 1734, in the 75th year of his chemiſtry, boldly attacking that horde of phyſical vermin, the alchemiſts, and inceſſantly darting the well-directed ſhafts of ſatire and criticiſm, againſt the abſurd prejudices and ignoble labours, which at that time diſparaged the ſcience of chemiſtry. He not only was inſtrumental in pulling down and demoliſhing a fabric erected with the moſt whimſical and groteſque materials, but he alſo poſſeſſed talents and ingenuity adequate to the rearing of a new ſyſtem, compiled, in ſome meaſure, from the veſtiges and fragments of former theories; a ſyſtem incomparably more rational and juſt than thoſe of his predeceſſors, and of conſiderable advantage to the ſubſequent progreſſive improvements which have taken place in that ſcience. In a word, he projected a thorough reform in chemiſtry; retaining however what appeared uſeful, and ſuperadding whatever he conceived to be neceſſary towards the eſtabliſhment of his new doctrines. On theſe principles aroſe his famous *ſyſtem of phlogiſton*, which, however variouſly modified, has preſerved its original character to the preſent day.

The following principles conſtitute the general outline of this ſyſtem:

There is a principle ſubſiſting in nature, which is the *ſole* and *excluſive* cauſe of heat and fire. This principle, *phlogiſton*, pervades all material bodies whatever, and indeed is a *conſtituent part* of the ſame; although it prevail in different proportions. Subſtances which poſſeſs a conſiderable quantity of it, are called *combuſtible* or *phlogiſtic* bodies. Theſe *only* are capable of producing heat, light, and fire. *Phlogiſton* appears to be a *ſubtile earth*, intimately combined with the *matter of fire*. By a *violent concuſſion of its parts* the motion of fire is produced; and on this occaſion the phlogiſton is partly decompoſed, and partly *volatilized*.—If both theſe phenomena take place, the *acceſs of air* will be indiſpenſably neceſſary; as, without it, the phlogiſton would be *fixed in the fire*, and *not decompoſed*;—of this, the ruſt of iron affords a proof, wherein the phlogiſton exiſts, in the higheſt degree of accumulation. There is *no ſpecific gravity* in this element, yet is it capable of rendering fixed bodies volatile. *Colours* are indebted to it for their exiſtence. To metals it imparts luſtre, coheſion, and ſolidity; all of which they loſe when deprived of phlogiſton, but recover by its acceſs. It is therefore an *eſſential* conſtituent in all metallic bodies; yet the precious metals do not appear to contain it*.

Such his uſeful life, and without doubt is one of the brighteſt luminaries, of which the annals of medicine can boaſt.

* Stahl's Guide to Metallurgy, including an Introduction to the Knowledge of the Subterranean, Mineral, and Metallic Bodies, together with their original Mixture, (in German,) 8vo Leipzig. 1720. Stahl's Reflections on the Diſpute reſpecting the Nature of Sulphur. 8vo. Halle. 1718.

Such is the outline of the ingenious ſyſtem created by Stahl. Conſidering the imperfect ſtate of chemiſtry in that age, it was a maſter-piece of his profound genius, a phenomenon highly conducive to the progreſs of phyſical knowledge, and a glorious precurſor to the actual improvement which this ſcience has ſince attained. Stahl it was, who gave a ſtable point of gravitation to chemiſtry; till then toſſed about, as it were, by the blind operations of ſuperſtitious fanatics and jugglers. He reduced the complicated train of phenomena to a regular ſeries, eſtabliſhed on ſcientific principles; he ſhed the light of day on the obſcure laboratories of the alchemiſts, and diſperſed the miſts of their hyperphyſical reveries, which were moreover expreſſed in the moſt unintelligible jargon. Henceforth chemiſtry aſſumed a new and more advantageous form, and has ever ſince juſtly claimed the appellation of a ſcience, which until that period could not be granted to it with propriety. Many of the learned who were not totally immerſed in the ſpeculations of alchemy, applied themſelves with laudable zeal to the further elucidation and improvement of his new doctrine, and Stahl now enjoys the acknowledged honour of being conſidered as the founder of *ſcientific chemiſtry*.

It was to be expected however, that the defects and imperfections of this ſyſtem would be ſoon diſcovered, in an age of progreſſive cultivation of the ſciences. Many phenomena could not be ſatisfactorily explained, others were entirely overlooked, by the author of the new theory of phlogiſton. Of this nature are the abſorption of reſpirable air during combuſtion and calcination; the increaſe of weight acquired by bodies during that operation; the eſcape of vital air in the reduction of mercury when performed in cloſe veſſels; the formation of carbonic acid or fixed air; the decompoſition and reproduction of water; together with a number of other phenomena which, in Stahl's ſyſtem, were couched in problems, the ſolution of which was deemed impoſſible.

Many zealous and acute chemiſts have endeavoured to ſupply theſe defects, and to render this ſyſtem more deſerving of that name; others have rejected it *in toto*, and directed their ſpeculations purpoſely to frame another theory, which might more ſatisfactorily account for all the unexplained phenomena.

From this happy ſectariſm in chemiſtry, two diſtinguiſhed parties have taken their riſe, that of the advocates for phlogiſton, who appear obſtinately determined not to relinquiſh their favourite hypotheſis; and that of the antiphlogiſtic ſchool, which has daily and rapidly increaſed in numbers, and in which the exiſtence of phlogiſton was, and is, boldly denied and ridiculed. With the well-known philoſophic chemiſt of France, the immortal, though though unfortunate Lavoisier*, at their head, the latter party has at length prevailed; yet not without a long and fevere conteft, which has terminated in an almoft complete victory over the difciples of Stahl. The difputed point was canvaffed with uncommon ardour by men of fuperior merit, on both fides of the queftion, and the conflict was maintained for feveral years, with various and very unequal fuccefs. The ground-work of this ftructure, the *ci-devant* phlcgifton, has been fhifted and modified in a thoufand different ways and forms. At one time it was not permitted to penetrate through veffels; at another time they allowed it a free paffage to and from them. Some pronounced it *neutral* † with refpect to its fpecific gravity; fome maintained that this gravity was *negative* (*a*); with fome it was a fubftance of an unknown bafis (*b*); with others it was a combination of the elements of air and fire (*c*); or inflammable air itfelf (*d*); again with others, it was confidered as pure light (*e*); and laftly, it was faid to confift of the matters of heat and light in a combined ftate (*f*). In fhort, it was a Proteus, who affumed every poffible figure, and could adapt himfelf to any form whatever.

In confequence of thefe ill-according opinions, and to avoid the difgrace of appearing to favour doctrines fo irreconcileable, a new fyftem of chemiftry was framed in Germany, which originated chiefly from the ideas of three celebrated chemifts, Wiegleb, Gren, and Westrumb, and which was ftyled the *improved* phlogiftic fyftem. The following are its principal tenets: The matter of *light* and *heat* in a *ftate of combination*, exhibit *phlogifton*. During the inflammation of combuftible bodies, thefe matters are fet at liberty or difengaged, either fingly or in a combined ftate; hence arife light, heat, and fire. This difengagement however, can only take place, when the phlogifton acquires activity by *communication* with the atmofpheric air, or with that conftituent part which renders it fit for the refpiration of animals. This elaftic matter, termed *pure* or *dephlogifticated air*, confifts of *water* and *matter of heat*; it is of all fubftances

* It is impossible to pronounce the name of this illustrious Frenchman, without feeling that degree of sympathy and indignation, which his untimely and shocking end must excite in the bosom of every friend of science, and persecuted innocence. Lavoisier was among the victims of fanatical, and, as some will maintain it, aristocratical vengeance: he suffered under the guillotine, in 1794, which was then totally at the command of that sanguinary monster, Robespierre, of infamous memory. We sincerely deplore the loss of Lavoisier; a man more to be regretted than any other that has fallen since the beginning of the struggle.

† This was the theory of Stahl, and his strict adherents.

(*a*) Gren. (*b*) Scheele and Crawford. (*c*) Baumé. (*d*) La Metherie. (*e*) Maquer. (*f*) Wieglcb and Gren.

ſubſtances that which is the leaſt ſaturated with phlogiſton, and conſequently it manifeſts a conſiderable power of attraction for phlogiſton. But by this combination, it is deprived of its property of ſupporting the life of animals, and producing the combuſtion of bodies. It is changed into phlogiſticated air, or that ſpecies of it, in combination with which it forms the atmoſphere. In this ſtate, however, it is reduced to an incomparably ſmaller volume and weight, than it before poſſeſſed; becauſe phlogiſton being a body of a *negative ſpecific gravity*, it poſſeſſes the power of rendering poſitively lighter all ſuch ſubſtances as enter into combination with it.

From theſe aſſumed properties of phlogiſton it has been maintained by its advocates, that the increaſe of metals during calcination originates from the ſame cauſe, but that the metallic calxes ſuffer a dimunition of weight in their regeneration; as, in the former caſe, they loſe that ſubſtance which rendered them abſolutely lighter than their own earthy baſe; and, in the latter, this baſe again recovers the principle of ſpecific gravity, and they muſt therefore neceſſarily loſe a part of their weight.

*Inflammable air* conſiſts of *water* combined with *phlogiſton*, and rendered elaſtic by matter of heat. It will always and neceſſarily ariſe, when water enters into contact with thoſe bodies which it has power to deprive of their phlogiſton. But in theſe caſes the water, which is an *elementary ſubſtance*, is not decompoſed, as it only enters into a new combination, and changes its form.—Water therefore muſt always be produced in the exploſion of this air; becauſe thoſe ſubſtances become diſengaged, which rendered the latter inflammable and gaſiform, that is to ſay, an elaſtic fluid.

The *carbon*, or charcoal, is a compounded body which conſiſts of *phlogiſton* and *aërial acid*. The latter therefore is no new production, but rather an eduction.

*Sulphur* and *phoſphorus* conſiſt of their own *peculiar acids* and of *phlogiſton*; by depriving them of the latter, the former become diſengaged, and appear in a free or uncombined ſtate.

Whatever advantages this modification of the phlogiſtic ſyſtem ſeemed to poſſeſs, when compared with the doctrines of Stahl, it was found to be in many reſpects, unſatisfactory, and ill-qualified to reſiſt the attacks inceſſantly urged by the opponents of phlogiſton. The hypotheſis reſpecting negative ſpecific gravity, although ingeniouſly contrived, was particularly untenable, and its advocates were under the neceſſity of ſoon retracting it. The ſingular opinion of Westrumb, that the increaſe of weight, in bodies which have undergone the proceſs of calcination, muſt be aſcribed to the *hygroſcopic*

*water,*

*water*, was likewiſe eaſily confuted; ſo that the principal pillars of the ſyſtem being ſhaken to their very foundation, the hypothetical notion of phlogiſton was thenceforth conſidered as fairly overthrown.

Much better founded is the antiphlogiſtic theory, which is now very generally adopted, and of which MAYOW *, an illuſtrious phyſician of this country, had ſketched out the firſt idea, more than a century ago. But unnoticed by his cotemporaries, he either lived or died too early to conſolidate and extend his opinion. Nay, inſenſible of his rare merit, as an original genius in the ſcientific department of phyſics, ungrateful poſterity would perhaps have conſigned his memory to oblivion, had not an acute modern chemiſt, his countryman, Dr. SCHERER †, of Jena, reſcued his ſublime opinions from his neglected writings, and thus erected a proud trophy

* No chemist or philosopher of former ages deserves more respectful mention than MAYOW. He published *five* remarkable Tracts which were printed at the Hague, in 1681, and dedicated to Henry Coventry, first secretary of state to Charles II. The first is on *salt-petre and its nitro-aereal spirit*, water-spouts, lightning, &c.; the second is on *respiration*; the third is on the *respiration of the fœtus in the womb and in the egg*; the fourth is on *muscular motion*; and the fifth, on *the rickets*. Whoever examines his fifth plate, will see, that inverted jars of air, and animals within them, were used in his experiments; and that he knew the use of the double convex lens, to set fire to substances confined in given quantities of air. Such a man, we think, should be called an *experimental philosopher*; and the more so, as some parts of his apparatus appear to be as well contrived as those of the present day.

Mayow published truths, for which the minds of his cotemporaries were not prepared. His luminous researches were given to the world about a century too soon. They were loſt---and the finding them, and making known their contents, constitute one of the many claims of Dr. Beddoes to public esteem.

*Medical Repository*, Vol. II. p. 194.

† This promising young chemist has acquired considerable and well-merited celebrity, partly by the ingenious chemical lectures he has, till lately, given in the University of Jena, where he was admitted public lecturer, in the twenty-second year of his age, and partly by several excellent publications in chemistry, chiefly adapted to the capacities of popular readers, or calculated to afford an elementary view of the antiphlogistic theory. In winter, 1797—8, he visited England and Scotland, where I had the good fortune of becoming personally acquainted with him. He travelled by appointment of the reigning Duke of Saxe-Weimar, a prince of the most accomplished literary talents, and a true Mecænas of neglected merit. Scherer now enjoys a handsome salary, in the capacity of Counsellor of mines to the Duke, and Superintendent of the mineral works established in the Duchy of Weimar. Having exerted himself for three or four years in the abovementioned University, to render his "*Experimental Courses of Chemistry*", equally interesting and useful to the students of all professions; and having met with very indifferent encouragement (on account of his not being *professor appointed with a salary*; so that, according to his own words, in winter 1796 he had only *sixteen* pupils, at a fee of about two guineas trophy to a name which is now juſtly intitled to immortality in the annals of chemiſtry. But, before this apotheoſis was beſtowed on the manes of Mayow, Lavoiſier, one of the moſt acute and inquiſitive men that France ever gave birth to, had ingeniouſly applied the conjectures of our countryman; and this with very ſingular ſucceſs. It is highly probable that the aphoriſms of Mayow, (after he had confirmed and eſtabliſhed them by the teſt of experience,) inſpired him with the idea, to conſtruct a new ſyſtem, which ſhould effectually and for ever ſuperſede that of phlogiſton. Gifted with an accompliſhed, penetrating and unprejudiced mind, Lavoiſier appears, in moſt points, to have cloſely conſulted, and as it were interrogated nature, in the formation of his admirable ſyſtem. The foundation of it is eſtabliſhed by facts founded on the moſt accurate experiments, and ſo ſyſtematically arranged, that the ſtudent of chemiſtry is equally captivated with the indications of his profound perſpicacity, and with the excellent and elegant manner in which he connects and illuſtrates his propoſitions. We ſhall here endeavour to exhibit the moſt ſimple outlines of Lavoiſier's "*New Syſtem of Chemiſtry*", and point out its excellence, by comparing it with others of a later date.

☞ *On account of the great variety of important articles which have come to hand, we are under the neceſſity of poſtponing the continuation of this intereſting eſſay to our next, and probably ſeveral ſubſequent numbers.*

guineas each, for a course of Lectures continued through *six months, two hours every day!*) we rejoice in his release from so unprofitable and ill-rewarded an undertaking. These difficulties, however, have not been without their concomitant good effects; for, during this period, Scherer employed great part of his leisure in the more minute investigation of various unsettled chemical subjects, and in publication of three different chemical works of uncommon merit, as elementary treatises. It was principally from these, he derived the greater part of his support, as he had been cruelly neglected by his wealthy relations, who originally educated him for the church. Thus, indeed, all his talents were called forth, and usefully exerted in the cause of a science, which is indebted to him for many sagacious hints and accurate illustrations—if not for original discoveries.

With respect to his merit in pointing out the extraordinary marks of original genius and talent, which were buried for a century in the writings of Mayow, we shall not attempt to decide whether he, or Dr. Beddoes, deserve the credit of having introduced Mayow to public notice and esteem.

For the literary engagements and pursuits of Dr. Scherer, we refer the reader to the notice given of his latest publications in our Journal, under the head *Medical and Physical Intelligence.*

