# Erratum

**Published:** 1799-04

**Authors:** 


——69, l. 11, for 'Jena,' read *Vienna;* and, consequently, the whole of the note concerning Dr. Scherer relates to another person of the same name, viz. *Dr. Scherer, the chemist of Jena.*

——70. In the penult paragraph of the note, the reader is requested to observe, that it was *Dr. Scherer, of Vienna*, and not the chemist of Jena, who first introduced *Mayow* to public notice in Germany.

