# Notice of Erratum, by Dr. Beddoes

**Published:** 1799-04

**Authors:** Thomas Beddoes

**Affiliations:** Clifton


### *To the Editors of the Medical and Phyſical Journal.*

GENTLEMEN,

IN an article of your firſt Number, in which the Author is pleaſed to ſpeak of me in relation to MAYOW, he confounds the two SCHERERS—one of whom wrote againſt Dr. FERRO, on the Uſe of Vital Air; and alſo publiſhed an Analyſis of Mayow;—while the other, the chemiſt of Jena, propoſed to tranſlate my Abridgment, (ſee the *Annales de Chimie*) but now intends to tranſlate Mayow, and to add an Appendix*. The former is uſually called Scherer of Vienna. Whether this Scherer, or myſelf, firſt drew notice towards Mayow, is a queſtion of total indifference to me.

THOMAS BEDDOES.

*Clifton, March,* 1799.

---

* *For a more particular account, on this ſubject, we muſt refer the Reader to the article,* "MEDICAL AND PHYSICAL INTELLIGENCE," *in this Number, page* 204.

